# The Treatment of Internet Gaming Disorder: a Brief Overview of the PIPATIC Program

**DOI:** 10.1007/s11469-017-9825-0

**Published:** 2017-11-13

**Authors:** Alexandra Torres-Rodríguez, Mark D. Griffiths, Xavier Carbonell

**Affiliations:** 1 Universitat Ramon Llull, C. Císter 34, 08022 Barcelona, Spain; 2 International Gaming Research Unit, Psychology Department, Nottingham Trent University, 50 Shakespeare Street, Nottingham, NG1 4FQ UK

**Keywords:** Internet addiction, Gaming addiction, Online videogames, Cognitive behavioral therapy, Manualized treatment, Internet Gaming Disorder

## Abstract

Over the last decade, there has been an increase in children and adolescents accessing psychology services regarding problematic use of online videogames. Consequently, providing effective treatment is essential. The present paper describes the design process of a manualized PIPATIC (Programa Individualizado Psicoterapéutico para la Adicción a las Tecnologías de la información y la comunicación) intervention program for 12- to 18-year-old adolescents with Internet Gaming Disorder. The design and application of the PIPATIC program integrates several areas of intervention structured into six modules: psychoeducational, treatment as usual, intrapersonal, interpersonal, family intervention, and development of a new lifestyle. The program’s goals are to reduce the addiction symptoms related to online videogames and to improve the well-being of adolescents. Preliminary findings suggest positive and encouraging effects.

Information and communication technologies (ICTs) have become fully integrated in people’s day-to-day lives. The increasing engagement in social networking, online videogame playing, and using smartphone applications has brought about new interaction styles. One group that often uses such online applications in both positive and negative ways are adolescents (Kuss and Griffiths 2012; Kuss and Griffiths 2017). On the positive side, online applications have been beneficial in research, science, medical, scholar, social networking, etc. On the negative side, excessive use may lead to negative consequences such as addictive behaviors among a small minority of users (Kowert et al. 2014; Williams et al. 2008).

## Online Videogames

The main objective of playing videogames is to entertain users via interactive systems that have now been developed across multiple platforms (e.g., personal computer, game consoles, mobile phones, and tablets). At present, most videogame applications have been developed with good online accessibility and accompanying social platforms (e.g., *Twitch*, *Steam*, and *Origin*). Consequently, there are hundreds of genres and sub-genres of online videogames. These genres include (i) massively multiplayer online role-playing games (MMORPGs), such as *World of Warcraft* (WoW); (ii) multiplayer online battle arena (MOBA) games, such as *League of Legends* (LoL); and (iii) first-person shooter (FPS) games such as action games like *Battlefield* (BT), *Counter Strike* (CS), and *Call of Duty* (CoD). There are also “social” videogames, such as *Angry Birds,* which are usually very simple and quick to play and have been developed to play via social networks and smartphones (Carbonell et al. 2016). At present, MOBA games are among the most popular. Its increasing popularity may be due to its structural characteristics such as the high level of competitiveness and free-to-play modes, and situational characteristics such as its high accessibility (Griffiths and Nuyens 2017). In spite of that, the MMORPGs are still popular with millions of players.

However, such classification may become less important due to the way new games are evolving. Newer games have begun to incorporate multiple genres and game modes simultaneously: action games (survival, shooter, platform, etc.), simulation games (vehicle, life, and construction simulation), role-playing games (MMORPGs), strategy games (MOBA, wargames, etc.), adventure games, sports games, cooperative games, and casual games. For example, *Day Z*, *Star Citizen*, and *Minecraft* are current online games with multiple genres and game modes. Furthermore, videogames are becoming more realistic and complete, offering users immersive playability.

There is also increasing convergence between online gaming and other online activities such as gambling, activities that are primarily based on positive and negative reinforcement. Other similar characteristics include the following: (i) high sociability, (ii) multiplayer features, (iii) potential for high immersion in virtual environments, (iv) creation of one’s own virtual identity and/or character development, and (v) possibility of infinite duration within a 24/7 environment (Griffiths and Nuyens 2017; King et al. 2010b; Wood et al. 2004). All five of these things can be found in online gambling.

Current models suggest that some structural characteristics of online video games have the capacity to contribute to the development of a problematic use and/or addictive behaviors leading to a deterioration in the self-control of behavior and quality of life (Carbonell et al. 2012; Fuster et al. 2016; Nuyens et al. 2016). To understand this relationship, it is important consider the problem from an eclectic and multidisciplinary perspective, including those working from a clinical mental health perspective.

## Clinical Psychology Perspective on Problematic Gaming

A good clinician will always try to understand the many aspects that may influence the acquisition, development, and maintenance of problematic online videogame use. Many researchers advocate a biopsychosocial model in explaining the interaction between different risk factors (e.g., Kelly 2004; Kuss and Griffiths 2012; Mazurek and Engelhardt 2013; Rehbein et al. 2010; Wittek et al. 2015; Yee 2006). Table 1 lists many of the main risk factors in the development of problematic online gaming.

**Table 1:** Selective important risk factors in addiction to online videogames

1) Biological factors • Vulnerability to addictions • Deficits in neurotransmitters • Psychiatric comorbidity: depression, anxiety, ADHD, ASD, etc. 2) Personality and psychological vulnerability factors • Immaturity • Emotional instability • Unconsolidated identity • Low self-esteem and indecision • Lack of self-control • Frustration • Low resilience • High sensation search • Deficit of social skills, inhibition and extreme shyness	3) Environmental factors • Family environment: conflictive, poor communication and affection, lack of supervision and family cohesion, etc. • School environment with low performance, demotivation, etc. • Poor social environment 4) Stress factors • Grief • Major crises • Drastic life changes 5) Structural factors

Online gaming addiction has been associated with (and may initiate) other comorbid mood disorders such as depression, stress, and anxiety disorders (Ferguson et al. 2011; King and Delfabbro 2014; Pontes et al. 2014). Consequently, videogame addiction in the form of Internet Gaming Disorder (IGD) was included in Section 3 of the latest (fifth) revision of the Diagnostic and Statistical Manual of Mental Disorders (DSM-5; American Psychiatric Association 2013). The clinical diagnosis of IGD comprises persistent and recurrent use of the Internet to engage in games, leading to clinically significant impairment or distress over a period of 12 months as indicated by five (or more) of the following criteria: (i) pre-occupation with Internet games; (ii) withdrawal symptoms when Internet gaming is taken away; (iii) tolerance or, in other words, the need to increase the time devoted to videogames; (iv) unsuccessful attempts to control participation in Internet games; (v) loss of interests in previous hobbies and entertainment as a result of, and with the exception of, Internet games; (vi) continued excessive use of Internet games despite knowledge of psychosocial problems; (vii) deceiving family members, therapists, or others regarding the amount of Internet gaming; (viii) use of Internet games to escape or relieve negative moods; and (ix) jeopardizing or losing a significant relationship, job, or education or career opportunity because of participation in Internet games. The criteria proposed by the APA ( 2013) appear to have adequate validity and diagnostic precision (Ko et al. 2014). Its prevalence, according to the American Psychiatric Association (2013), is between 1 and 7%. Part of this variation is due to the fact that different instruments are used in different cultural contexts and for different ages (Feng et al. 2017; Ferguson et al. 2011; Müller et al. 2015; van Rooij et al. 2014).

According to Griffiths, King, and Demetrovics (Griffiths et al. 2014), the inclusion of IGD in the DSM-5 provided the opportunity for consensus and unification in the field. However, there is still no consensus on many issues related to the DSM-5 diagnostic criteria for IGD, and its psychological, social, and health consequences still require further study (Griffiths et al. 2016b; Király et al. 2015; Kuss et al. 2016). The lack of consensus concerning diagnostic criteria makes the work of clinical psychologists and other therapists difficult. Consequently, there is a need to clearly define IGD, clarify its most controversial aspects, and expand the study of specialized IGD treatments.

## Psychological Treatment for Internet Gaming Disorder

Although the study of IGD has grown markedly in recent years (Griffiths et al. 2016a; Kuss and Griffiths 2012), evaluations of psychological treatments are still scarce. The most used treatment for online addictions appears to be cognitive behavioral therapy (CBT) based on the peer-reviewed literature (Greenfield 1999a, 1999b; Griffiths and Meredith 2009; Kaptsis et al. 2016; King et al. 2010b, 2011; Young 2007, 2013). Other treatments used include pharmacotherapy (methylphenidate and bupropion) and counseling (King et al. 2011). Most of the therapeutic recommendations are based on substance abuse treatment (Huang et al. 2010; King et al. 2011) including motivational interviewing techniques, stimulus control, learning appropriate coping responses, self-monitoring strategies, cognitive restructuring, problem-solving related to addiction, and withdrawal regulation techniques with exposure (Griffiths and Meredith 2009; King et al. 2010a; Young 2007). Furthermore, there has been little evaluation concerning the efficacy of different psychological interventions with children and adolescents (King et al. 2013; King and Delfabbro 2016).

With regard to clinical practice, therapies for IGD based on substance abuse treatment have a number of limitations. One of the most important is the high rate of comorbid disorders with IGD that could influence the effect of the treatment. Some of the most common disorders associated with IGD include anxiety disorders, mood disorders, behavioral disorders, autism spectrum disorder (ASD), attention deficit hyperactivity disorder (ADHD), and personality disorders (Ferguson et al. 2011; Gentile et al. 2011; Han et al. 2014; Kelleci and Inal 2010; Ko et al. 2006; Shapira et al. 2000). Most published IGD treatment studies have not taken into account these comorbidities; therefore, more integrated and comprehensive treatments taking these comorbid disorders into account would improve treatment outcomes for such individuals (Therien et al. 2014).

Consequently, there is both an empirical and clinical need to generate specialized, integrated, and comprehensive treatment protocols taking into account the specific characteristics of IGD along with its associated comorbid disorders. Furthermore, it is clinically important to develop treatments for adolescents. In recent years, psychiatric wards have reported an increase of consultations regarding adolescents with problematic use of cyber-technologies (Beranuy et al. 2012). On the other hand, the adolescent population regularly uses online video games and they also present with multiple psychological vulnerabilities that are intrinsic to this life stage.

As a result, the aim of the present paper describes a specialized and integrative treatment program based on the scientific and clinical need to design treatments specifically for the adolescent population with IGD. The treatment program provides essential and common factors concerning IGD and was developed for health professionals to utilize in the treatment of IGD in adolescence.

## Stage I: Designing and Developing the Treatment Program

To develop bespoke psychotherapy (in this case, for IGD), manuals in clinical practice must be followed to define guidelines and strategies from basic outlines in order to promote empirically supported treatments (Carroll and Nuro 2002). In the development and design of the PIPATIC program, there are common and essential aspects that should be included in any program evaluation report (Moscosoa et al. 2013; Schulz et al. 2010). The word “PIPATIC” is an acronym for “Programa Individualizado Psicoterapéutico para la Adicción a las Tecnologías de la información y la comunicación”. The English translation for this Spanish program is “Individualized psychotherapy program for addiction to information and communication technologies.”

The guidelines proposed by Carroll and Nuro (2002) were used for the IGD treatment presented in the present paper due to their rigor and scientific underpinnings. Carroll and Nuro divide the development of treatment manuals into three stages: (i) the development of the treatment and a pilot application, (ii) the design of the methodology with a controlled clinical trial to evaluate the efficacy of the treatment, and (iii) the evaluation of the application of treatment in different contexts and the relationship between their effectiveness and costs.

The goal of the newly developed PIPATIC program (Torres-Rodriguez and Carbonell 2017) is to offer specialized psychotherapy for adolescents with symptoms of IGD and comorbid disorders. This program comprises six therapeutic work modules, in turn comprising further specific sub-objectives. Following previous studies in order to ensure therapeutic change in patients, the duration of the program is six-months (22 weekly sessions each lasting 45 min) (Hansen and Lambert 2003; Kadera et al. 1996; Lambert et al. 1994; Seligman 1995). The composition of the modules and sessions is briefly outlined in Tables 2, 3, 4, 5, 6, 7, and 8. Additionally, the PIPATIC program includes two floating sessions that can be incorporated into the module of the therapist’s choosing, according to the needs of the patient. In this way, the set program offers some flexibility (Carroll and Nuro 2002; Therien et al. 2014).

**Table 2:** Module 1: psychoeducation and motivations (three sessions)

Sub-modules	Content	Psychological strategies	Intervention techniques and strategies
Motivational aspects	Motivational interviewing	Active listening of the problem Express empathy Framing the personal situation Working on patient resistance	Self-motivation reinforces Explore ethical and personal values Decisional balance
Goal setting	Definition of goals in order to address their problems, to generate the beginning of change, promoting organization and responsibility daily habits	Be an active role in the negotiations Confronting non-therapeutic goals Make initial requests to increase their responsibility and autonomy	Make a negotiated goal list with patient, family and psychologist Elaboration of goals and habits reminder cards Schedule with therapeutic habits
Psychoeducation with the adolescent	ICTs and videogames: maladaptive and adaptive use, benefits and disadvantages Perception of their reality	Guide and give self-observation instructions to explore the use and knowledge of the ICTs Generate awareness of the problem Use motivational techniques	Rhetorical-pragmatic techniques Self-registers through self-observation Negotiation techniques Individual Psychoeducation Motivational techniques
Family psychoeducation	ICTs and videogames: maladaptive and adaptive use, benefits and disadvantages	Solve doubts Contain and regulate anxieties	Hetero-observation by the family Family Psychoeducation First guidelines for rational use of ICTs

**Table 3:** Module 2: addictions treatment as usual adapted to IGD (five sessions)

Sub-modules	Content	Psychological strategies	Intervention techniques and strategies
Addiction stimulus control	Craving control Self-control capacity Irrational beliefs of self-control	Exemplify real situations of loss of control Contain the difficulties of self-observation Detection of inappropriate uses To find relations between emotions, cognition and conduct	Stimulus control: establishing a connection time Self-registers Stopping thinking Positive reinforcement Self-reinforcement Tolerance to the frustration techniques
Coping responses	Inadequate coping responses related to the addiction and low self-control	Guide the reflection about the relationship with the addictive behavior Behaviors and thoughts interpretation To promote new adaptive responses in discomfort situations	Training in alternative coping responses Self-instruction training
Cognitive restructuration	Cognitive distortions and irrational beliefs that interfere with behavior control	Detect and restructure cognitive distortions and irrational beliefs Therapeutic work based on rational-emotive behavior therapy (Ellis 1990) and cognitive therapy (Beck 1979) Exemplify and use metaphors	ABC (A-Activating Event; B-Belief; C-Consequence) model explanation ABC self-registration exercises Socratic debates Exercises related to the imagination of situations and behavioral testing
Problem solving related to addiction	Identify, define, and solve addiction-related problem situations	To guide insights about discomfort able situations and the consequences To teach adequately to deal with these situations without resorting to addictive behaviors such as avoidance, flight, or stimulation	Identification of problem situations, involved emotions and thoughts Role-playings of the conflictive real situations with its alternative responses List of coping thoughts
Exposition	Disappearance of craving led to inappropriate behavior	To guide an exposition process to transform the compulsive behavior into adaptively and controlled activity To guide the co-therapist in to exposure process	Imaginative exposure Co-therapist exposure Stopping thinking Remembering coping thoughts and responses, encourage self-observation, reinforcement and self-reinforcement techniques

**Table 4:** Module 3: intrapersonal (five sessions)

Sub-modules	Content	Psychological strategies	Intervention techniques and strategies
Identity	Different levels of identity: self-ideal, ideal of the self and self Self-knowledge through introspection Stable identity and identity regarding addiction	To facilitate and contain the expression of emotions To generate connections and global visions To ask reflective questions about identity To reinforce the process of personal maturation (solid personality and autonomy)	Activities related to de self-knowledge of own identity Reinforcement Timeline techniques
Self-esteem	The three concepts of the self (Rogers 1959): self-image, self-esteem and ideal-self	To give information about self-esteem To guide the change related himself description Stimulate positive and proactive thoughts	Self-affirmation and self-reinforcement Establishment and modification of psychic and physical self-image through different activities: incomplete phrases, self-assessment, analysis of situations related to the success
Emotional intelligence	To produce self-control Strategies of self-management and emotional regulation	Positive reinforce the patient’s progress To connect the lack of self-control and anxiety with the corresponding emotions To develop the insight and self-knowledge To highlight abilities and positive aspects of the person	Emotional intelligence techniques Breathing and relaxing techniques Emotional regulation techniques and activities Techniques related to tolerance discomfort *Mindfulness*
Emotional coping skills	Strategies to generate adaptive behaviors Self-management of anxiety and negative emotions	To guide in the correct emotional management To use real examples discussed throughout therapy	Training in emotional alternative responses Strategies for self-regulation and self-instruction
Problem solving	Skills and strategies for solving intrapersonal problems Self-management of conflict situations	To encourage the expression of anxieties and fears regarding conflicting vital situations of the adolescent To guide the development of adaptive responses related to everyday life	Deep understanding of the several conflicts Practice the new skills in front new everyday situations

**Table 5:** Module 4: interpersonal (two sessions)

Sub-modules	Content	Psychological strategies	Intervention techniques and strategies
Communication	Interpersonal communication Communication styles	To detect if there is any negative communication style To generate a successful communication with other people	Psychoeducation about verbal and nonverbal communication Role-playings Activities related to communication skills
Assertiveness	Assertiveness Basic human rights	To work on building an assertive belief system To increase the insight, the empathy, and introspection abilities	Activities related to assertiveness
Answering styles	Answering styles Emotional, behavioral and communicational repetitive patterns related to answering styles Answering styles and consequences	To detect of the pattern of response style according to verbal and nonverbal communication To encourage the reflect about consequences of different styles To promote connections between answering styles and certain situations	Express and communicate Role-playing To comfort problems in an assertive way: to express, to point out the moment, to characterize, to adapt the feelings, to emphasize he cooperation, to limit, to postpone a conversation, to empathize, to defend one’s feelings, a scratched disc, etc.

**Table 6:** Module 5: family (three sessions)

Sub-modules	Content	Psychological strategies	Intervention techniques and strategies
Family communication	Family communication Communication styles at home Adaptive family communication	To contain negative emotions and anxieties related to family conflicts To detect communicational mistakes in the family To promote new communication skills in the family	Active listening Activities related to adaptive communication styles in the family Application of the contents daily Other specific activities
Limits establishment	Family limits Establishment of news family limits Family behaviors	To guide the identification of family behaviors and boundaries To establish decisions and new limits through negotiation strategies	Specific activates related to this sub-module, e.g., negotiation techniques, remembering rules without criticizing the others, the use of consequences instead of punishments
Bonds establishments	Affective bounds: insecure or ambivalent, avoidant, disorganized and secure	To detect the type of family bond To establish a new adaptive bond To improve family bonds To moderate the joint effort decisions To contain negative aspects of the therapy family	Psychoeducation related to affective bonds Role-playing techniques with the family Specific homework to improve new family skills and keep learnings Specific activities related to showing affection

**Table 7:** Module 6: creation of new lifestyle (two sessions)

Sub-modules	Content	Psychological strategies	Intervention techniques and strategies
Observations related to the improvements	Achieved goals Achieved new skills	To highlight the positive changes and new adaptive abilities To give new strategies to keep the changes during the time	Comparatives self-registrations Observations related to personal changes and new skills Balance between the previous and the current situation
Alternative activities	To generate new alternative activities to previous ones	To promote the reflection of the adolescent about activities to develop next To promote useful, positive and social activities	Developing a list about possible new activities that generate well-being and happiness
Relapse prevention	Relapse prevention	To explore current emotions To point out risk situations To provide specific ideas and resources to avoid the relapses	Anticipation strategies to prevent risk situations and relapses Activities and relapse prevention material

**Table 8:** Treatment conclusion and three-months follow-up

	Content	Psychological strategies	Intervention techniques and strategies
Conclusion of the treatment	Farewell	Conclusion setting To contain anxieties	Brief review of prevention relapse techniques Encourage the patient about its own recourses and the new life stage
Post-evaluation	Application of instrument battery	To guide the process	Correction and evaluation of instruments used
Follow-up	Strengthening processes and learning related to the treatment Follow-up visit	Assessing patient’s improvement Begin a process of detachment with the therapist To promote self-efficacy in the patient Address possible relapse risks	Supportive strategies Reinforcement of the self-efficacy Learnings generalizations

One of the main psychological treatments used in the PIPATIC program is CBT given its empirically supported efficacy (Hofmann et al. 2012; Nathan 2015). CBT therapies are commonly used and are effective in the treatment of addictive disorders (Du et al. 2010; King et al. 2012; Li and Wang 2013; Wölfling et al. 2014; Young 2007, 2013). However, the use of multiple psychotherapeutic strategies is considered more effective than one unique perspective (Dong and Potenza 2014; Orzack et al. 2006; Shek et al. 2009; Therien et al. 2014). Consequently, other eclectic and integrative treatment perspectives additional to CBT were included within the PIPATIC program such as motivational interviewing and psychoeducation techniques (see Table 2), strategies derived from person-centered therapy, self-regulation and maturation strategies for adolescents (see Table 4), family therapy (see Table 6), and solution-focused therapy (see Tables 4 and 7).

The intervention, based on a cognitive behavioral approach, employs cross-cutting techniques and resources common in psychotherapy (Hofmann and Barlow 2014; Kleinke 1994; Laska et al. 2014) including empathy, active and reflexive listening, acceptance, trust, intermediate degree of directivity, paraphrasing, clarification, synthesis, confrontation, interpretation, feedback, promoting abilities, promoting responsibility, encouraging a feeling of self-efficacy, raising insight, and promoting a therapeutic alliance.

At the same time, adolescence is considered to be a vulnerable stage of life, defined by characteristics that generate a high risk of addictive behavior and/or other psychological disorders (Masten and Garmezy 1985; Steinhausen and Metzke 2001). For that reason, the strategies and techniques incorporated into the PIPATIC program have been adapted for adolescents. Consequently, the PIPATIC program is an integrative psychotherapy treatment intervention that focuses on multiple critical areas of the individual.

## Stage II: Designing Application Methodologies

The second phase of program development focuses on the delimitation of methodologies for the application and effectiveness of the PIPATIC program. The CONSORT (Consolidated Standards of Reporting Trials) international guide (Schulz et al. 2010) was used to design the program. The CONSORT methodologies guarantee quality experimental or quasi-experimental studies: (i) design (clinical trial with randomization to avoid bias), (ii) dependent and independent variables and assessment instruments, (iii) inclusion and exclusion criteria, (iv) aims and hypothesis, (v) method, and (vi) ethics.

## PIPATIC Program

### Target Population

The target population of the PIPATIC program is the adolescents aged 12 to 18 years old.

### Clinical Objectives

The main aim of the program is to provide specialized psychological assistance for adolescents with IGD. The clinical objectives are as follows: (i) to gain adaptive (rather than maladaptive) use of videogames and ICTs; (ii) to enable therapeutic change to different patient profiles; and (iii) to treat adolescents multidimensionally, attending to intrapersonal needs (including the comorbid disorders), interpersonal needs, family needs, and educational/occupational needs.

### Summary of the PIPATIC Manual

The PIPATIC program was designed as a clinical manual comprising six modules with specific sub-modules. Each module includes several psychological techniques in addition to cross-psychotherapeutic strategies. The program is six month duration comprising twenty-two 45-minute weekly sessions each for both individuals and families. The PIPATIC intervention is an individual [person-to-person] therapy carried out by a qualified clinical psychologist. Before the commencement of treatment, several sessions are carried out with the following objectives: (i) to assess the inclusion and exclusion criteria, (ii) to talk to the patient and family and address any concerns they may have about the program, and (iii) to complete the first pre-treatment evaluation.

Tables 2, 3, 4, 5, 6, 7, and 8 summarize all the modules belonging to the PIPATIC program. The tables outline the main content, the psychological strategies used within the module, and the intervention techniques and strategies of every module. In addition, all the modules include therapeutic homework to strengthen the establishment related to the therapeutic changes required to help change everyday behavior. In the PIPATIC program, the direct collaboration of family in the treatment program is essential to make progress. It is uncommon for adolescents to ask for therapeutic help on their own (Griffiths 2015), and it is usually the adolescent’s family who ask for therapeutic care or psychological treatment (King et al. 2010b).

The efficacy of the treatment program shows excellent promise based on data collected on the 17 individuals that have undergone the program to date (Torres-Rodríguez and Carbonell, 2015, 2017; Torres-Rodríguez, Griffiths and Carbonell, 2017). These preliminary results of the PIPATIC program pilot application demonstrate a reduction of time spent playing videogames, a reduction in IGD-related symptoms and comorbid symptoms, and improvement in multiple important areas in day-to-day functioning (e.g., interpersonal and intrapersonal functioning, family functioning, and educational/occupational functioning). Furthermore, compared to other treatments for IGD and Internet addiction published in the peer-reviewed literature, the PIPATIC program is arguably one of the most comprehensive treatment intervention for Internet and online videogame addiction (see Table 9 for an in-depth comparison between studies published in the empirical clinical literature).

**Table 9:** Comparison between PIPATIC program and other relevant treatment programs

Psychological interventions related to technologies addictions	*N* = sample	Motivational interviewing	Setting aims	Psychoeducation	Self-observation and time organization	Stimulus control	Coping strategies	Cognitive restructuration	Withdrawal and exposure	Identity and self-esteem	Emotional intelligence	Social skills	Family therapy	Lifestyle management	Comorbid disorders treatment	Relapse prevention	Follow-up
PIPATIC	17																
Díaz et al. 2008	–																
Dong and Potenza 2014	–																
King et al. 2010a	–																
Young 2013 (CBT-IA)	128																
Li and Wang 2013 ^a^	14																
Kim 2008 ^a^	25																
Su et al. 2011	65																
Shek et al. 2009 ^b^	59																
Marco and Chóliz 2014	1																

present,  present with some limitations,  not present

^a^Group therapy

^b^Individual and group therapy (mixed)

## Conclusions

The present paper described the development of the PIPATIC program, a contemporary and integrative treatment based on CBT for the specialized treatment of adolescents with IDG. Its design is based on a theoretical robust empirical base, on previous treatment studies, and on a combination of perspectives and psychotherapeutic strategies that have proven to be effective on adolescents across different disorders (Du et al. 2010; Fang-ru and Wei 2005; Han et al. 2015; King et al. 2012; Nathan 2015; Winkler et al. 2013; Young 2009, 2013).

At present, the efficacy of the program appears very promising but due to the time-intensive nature of the intervention, only 17 clients have completed treatment to date. As with any other treatment or manualized program, there are a number of limitations. The program was designed focusing on a very specific population (i.e., adolescents with IGD). The intervention has a specific rigidity because it must follow or adhere to specific methodological standards for its application and evaluation. Occasionally, such rigidity is difficult to adapt to the client’s particular needs. Due to this, and to solve the “stiffness” of the treatment, the modules could be applied independently and in different order to adapt to the most urgent need that the client has. Despite these limitations, the preliminary results suggest the program is efficacious.

Future plans include (i) studying the effectiveness of PIPATIC treatment comparing the participants of this treatment with other participants receiving treatment as usual for addictions; (ii) comparing the data already collected in relation to pre-treatment, middle-treatment, post-treatment, and three-months follow-up; and (iii) analyzing various cases of different ages, clinical profiles, game genre, and context to confirm the efficacy of findings in other populations and environments. It is expected that the PIPATIC program will continue to be an effective and pioneering integrative treatment for problematic use and addictive behaviors related to online videogames as well as concomitant pathologies. The present paper attempts to provide an orientation and update for health professionals in this field who want to treat adolescents for IGD.

## References

[CR1] American Psychiatric Association (2013). DSM-5: diagnostic and statistical manual of mental disorders.

[CR2] Beck AT (1979). Cognitive therapy and the emotional disorders.

[CR3] Beranuy M, Carbonell X, Griffiths M (2012). A qualitative analysis of online gaming addicts in treatment. International Journal of Mental Health and Addiction.

[CR4] Carbonell X, Fúster H, Chamarro A, Oberst U (2012). Adicción a internet y móvil: Una revisión de estudios empíricos españoles. Papeles Del Psicólogo.

[CR5] Carbonell X, Torres-Rodríguez A, Fuster H, Echerburua E (2016). El potencial adictivo de los videojuegos. Abuso de Internet: ¿antesala para la adicción al juego de azar on-line?.

[CR6] Carroll KM, Nuro KF (2002). One size cannot fit all: a stage model for psychotherapy manual development. Clinical Psychology: Science and Practice.

[CR7] Díaz R, Beranuy M, Oberst U (2008). Terapia de la adicción a internet y video-juegos en niños y adolescentes. Revista de Psicoterapia.

[CR8] Dong G, Potenza MN (2014). A cognitive-behavioral model of Internet gaming disorder: theoretical underpinnings and clinical implications. Journal of Psychiatric Research.

[CR9] Du Y, Jiang W, Vance A (2010). Longer term effect of randomized, controlled group cognitive behavioral therapy for Internet addiction in adolescent students in Shanghai. The Australian and New Zealand Journal of Psychiatry.

[CR10] Ellis A, Dryden W, DiGiuseppe R (1990). Special features of rational-emotive therapy. A primer on rational-emotive therapy.

[CR11] Fang-ru Y, Wei H (2005). The effect of integrated psychosocial intervention on 52 adolescents with internet addiction disorder. Chinese Journal of Clinical Psychology.

[CR12] Feng W, Ramo DE, Chan SR, Bourgeois JA (2017). Internet gaming disorder: trends in prevalence 1998–2016. Addictive Behaviors.

[CR13] Ferguson C, Coulson M, Barnett J (2011). A meta-analysis of pathological gaming prevalence and comorbidity with mental health, academic and social problems. Journal of Psychiatric Research.

[CR14] Fuster H, Carbonell X, Pontes HM, Griffiths MD (2016). Spanish validation of the Internet Gaming Disorder (IGD-20) Test. Computers in Human Behavior.

[CR15] Gentile D, Choo H, Liau A, Sim T, Li D, Fung D, Khoo A (2011). Pathological video game use among youths: a two-year longitudinal study. Pediatrics.

[CR16] Greenfield DN (1999). Virtual addiction: help for netheads, cyberfreaks, and those who love them.

[CR17] Greenfield, D. N. (1999b). Psychological characteristics of compulsive internet use: A preliminary analysis. *Cyberpsychology & Behavior, 2*(5), 403–412. 10.1089/cpb.1999.2.403. 10.1089/cpb.1999.2.403 19178212

[CR18] Griffiths MD (2015). Problematic technology use during adolescence: why don’t teenagers seek treatment?. Education and Health.

[CR19] Griffiths MD, Meredith A (2009). Videogame addiction and its treatment. Journal of Contemporary Psychotherapy.

[CR20] Griffiths, M. D., & Nuyens, F. (2017). An overview of structural characteristics in problematic video game playing. *Current Addiction Reports*, 272–283. 10.1007/s40429-017-0162-y. 10.1007/s40429-017-0162-y PMC5554268 28845381

[CR21] Griffiths MD, King DL, Demetrovics Z (2014). DSM-5 Internet gaming disorder needs a unified approach to assessment. Neuropsychiatry.

[CR22] Griffiths, M. D., Kuss, D. J., Billieux, J., & Pontes, H. M. (2016a). The evolution of Internet addiction: a global perspective. *Addictive Behaviors, 53*, 193–195. 10.1016/j.addbeh.2015.11.001. 10.1016/j.addbeh.2015.11.001 26562678

[CR23] Griffiths, M. D., van Rooij, A. J., Kardefelt-Winther, D., Starcevic, V., Király, O., Pallesen, S., … Demetrovics, Z. (2016b). Working towards an international consensus on criteria for assessing internet gaming disorder: a critical commentary on Petry et al. (2014). *Addiction, 111*(1), 167–175. 10.1111/add.13057. 10.1111/add.13057 PMC5699464 26669530

[CR24] Han DH, Lee YS, Shi X, Renshaw PF (2014). Proton magnetic resonance spectroscopy (MRS) in on-line game addiction. Journal of Psychiatric Research.

[CR25] Han D, Kim SM, Lee YS, Renshaw PF (2015). The effect of family therapy on the changes in the severity of on-line game play and brain activity in adolescents with on-line game addiction. Psychiatry Research: Neuroimaging.

[CR26] Hansen NB, Lambert MJ (2003). An evaluation of the dose-response relationship in naturalistic treatment settings using survival analysis. Mental Health Services Research.

[CR27] Hofmann SG, Barlow DH (2014). Evidence-based psychological interventions and the common factors approach: the beginnings of a rapprochement?. Psychotherapy.

[CR28] Hofmann SG, Asnaani A, Vonk IJJ, Sawyer AT, Fang A (2012). The efficacy of cognitive behavioral therapy: a review of meta-analyses. Cognitive Therapy and Research.

[CR29] Huang X, Li M, Tao R (2010). Treatment of Internet addiction. Current Psychiatry Reports.

[CR30] Kadera SW, Lambert MJ, Andrews AA (1996). How much therapy is really enough?: a session-by-session analysis of the psychotherapy dose-effect relationship. Journal of Psychotherapy Practice and Research.

[CR31] Kaptsis D, King DL, Delfabbro PH, Gradisar M (2016). Withdrawal symptoms in internet gaming disorder: a systematic review. Clinical Psychology Review.

[CR32] Kelleci M, Inal S (2010). Psychiatric symptoms in adolescents with Internet use: comparison without Internet use. CyberPsychology, Behavior, and Social Networking.

[CR33] Kelly RV (2004). Massively multiplayer online role-playing games: the people, the addiction and the playing experience.

[CR34] Kim JU (2008). The effect of a R/T group counseling program on the Internet addiction level and self-esteem of Internet addiction university students. International Journal of Reality Therapy.

[CR35] King DL, Delfabbro PH (2014). Internet gaming disorder treatment: a review of definitions of diagnosis and treatment outcome. Journal of Clinical Psychology.

[CR36] King, D. L., & Delfabbro, P. H. (2016). Features of parent-child relationships in adolescents with Internet Gaming Disorder. *International Journal of Mental Health and Addiction*. 10.1007/s11469-016-9699-6.

[CR37] King D, Delfabbro P, Griffiths M (2010). Cognitive behavioral therapy for problematic video game players: conceptual considerations and practice issues. Journal of Cyber Therapy and Rehabilitation.

[CR38] King D, Delfabbro P, Griffiths M (2010). Video game structural characteristics: a new psychological taxonomy. International Journal of Mental Health and Addiction.

[CR39] King D, Delfabbro P, Griffiths M, Gradisar M (2011). Assessing clinical trials of Internet addiction treatment: a systematic review and CONSORT evaluation. Clinical Psychology Review.

[CR40] King DL, Delfabbro PH, Griffiths MD, Gradisar M (2012). Cognitive-behavioral approaches to outpatient treatment of internet addiction in children and adolescents. Journal of Clinical Psychology.

[CR41] King DL, Delfabbro PH, Griffiths MD (2013). Trajectories of problem video gaming among adult regular gamers: an 18-month longitudinal study. CyberPsychology, Behavior and Social Networking.

[CR42] Király O, Griffiths MD, Demetrovics Z (2015). Internet Gaming Disorder and the DSM-5: Conceptualization, debates, and controversies. Current Addiction Reports.

[CR43] Kleinke CL (1994). Common principles of psychotherapy.

[CR44] Ko CH, Yen JY, Chen CC, Chen SH, Wu K, Yen CF (2006). Tridimensional personality of adolescents with internet addiction and substance use experience. The Canadian Journal of Psychiatry.

[CR45] Ko C-H, Yen J-Y, Chen S-H, Wang P-W, Chen C-S, Yen C-F (2014). Evaluation of the diagnostic criteria of Internet gaming disorder in the DSM-5 among young adults in Taiwan. Journal of Psychiatric Research.

[CR46] Kowert R, Festl R, Quandt T (2014). Unpopular, overweight, and socially inept: reconsidering the stereotype of online gamers. CyberPsychology, Behavior and Social Networking.

[CR47] Kuss DJ, Griffiths MD (2012). Internet gaming addiction: a systematic review of empirical research. International Journal of Mental Health and Addiction.

[CR48] Kuss DJ, Griffiths MD (2012). Online gaming addiction in children and adolescents: a review of empirical research. Journal of Behavioral Addictions.

[CR49] Kuss, D. J., & Griffiths, M. D. (2017). Social networking sites and addiction: ten lessons learned. 10.3390/ijerph14030311. 10.3390/ijerph14030311 PMC5369147 28304359

[CR50] Kuss, D. J., Griffiths, M. D., & Pontes, H. M. (2016). Chaos and confusion in DSM-5 diagnosis of Internet Gaming Disorder: issues, concerns, and recommendations for clarity in the field. *Journal of Behavioral Addictions*, 1–7. 10.1556/2006.5.2016.062. 10.1556/2006.5.2016.062 PMC5520132 27599673

[CR51] Lambert MJ, Shapiro DA, Bergin AE, Garfield SL, Bergin AE (1994). The effectiveness of psychotherapy. Handbook of psychotherapy and behavior change.

[CR52] Laska KM, Gurman AS, Wampold BE (2014). Expanding the lens of evidence-based practice in psychotherapy: a common factors perspective. Psychotherapy.

[CR53] Li H, Wang S (2013). The role of cognitive distortion in online game addiction among Chinese adolescents. Children and Youth Services Review.

[CR54] Marco C, Chóliz M (2014). Tratamiento cognitivo-conductual de la adicción a videojuegos de rol online. Anales de Psicologia.

[CR55] Masten AS, Garmezy N, Masten AS, Garmezy N (1985). Risk, vulnerability, and protective factors in developmental psychopathology. Advances in clinical child psychology.

[CR56] Mazurek MO, Engelhardt CR (2013). Video game use in boys with autism spectrum disorder, ADHD, or typical development. Pediatrics.

[CR57] Moscosoa SC, Chaves SS, Vidal MP, Anguera MT (2013). Reporting a program evaluation: needs, program plan, intervention, and decisions. International Journal of Clinical and Health Psychology.

[CR58] Müller KW, Janikian M, Dreier M, Wölfling K, Beutel ME, Tzavara C (2015). Regular gaming behavior and internet gaming disorder in European adolescents: results from a cross-national representative survey of prevalence, predictors, and psychopathological correlates. European Child & Adolescent Psychiatry.

[CR59] Nathan PE (2015). A guide to treatments that work.

[CR60] Nuyens F, Deleuze J, Maurage P, Griffiths MD, Kuss DJ, Billieux J (2016). Impulsivity in Multiplayer Online Battle Arena Gamers: preliminary results on experimental and self-report measures. Journal of Behavioral Addictions.

[CR61] Orzack MH, Voluse AC, Wolf D, Hennen J (2006). An ongoing study of group treatment for men involved in problematic Internet-enabled sexual behavior. CyberPsychology & Behavior.

[CR62] Pontes HM, Király O, Demetrovics Z, Griffiths MD (2014). The conceptualisation and measurement of DSM-5 Internet Gaming Disorder: the development of the IGD-20 Test. PLoS One.

[CR63] Rehbein F, Psych G, Kleimann M, Mediasci G, Mößle T (2010). Prevalence and risk factors of video game dependency in adolescence: results of a German nationwide survey. CyberPsychology, Behavior, and Social Networking.

[CR64] Rogers, C. (1959). *A theory of therapy, personality and interpersonal relationships as developed in the client-centered framework*. In S. Koch (Ed.), Psychology: A study of a science. vol. 3: formulations of the person and the social context. New York: McGraw Hill.

[CR65] Schulz KF, Altman DG, Moher D, Group, C (2010). CONSORT 2010 statement: updated guidelines for reporting parallel group randomized trials. Annals of Internal Medicine.

[CR66] Seligman ME (1995). The effectiveness of psychotherapy: the consumer reports study. American Psychologist.

[CR67] Shapira NA, Goldsmith TD, Keck PE, Khosla UM, McElroy SL (2000). Psychiatric features of individuals with problematic internet use. Journal of Affective Disorders.

[CR68] Shek D, Tang V, Lo CY (2009). Evaluation of an internet addiction treatment. Adolescence.

[CR69] Steinhausen HC, Metzke CW (2001). Risk, compensatory, vulnerability, and protective factors influencing mental health in adolescence. Journal of Youth and Adolescence.

[CR70] Su W, Fang X, Miller JK, Wang Y (2011). Internet-based intervention for the treatment of online addiction for college students in China: a pilot study of the Healthy Online Self-helping Center. CyberPsychology, Behavior and Social Networking.

[CR71] Therien P, Lavarenne SA, Lecomte T (2014). The treatment of complex dual disorders: clinicians’ and service users’ perspectives. Journal of Addiction Research and Therapy.

[CR72] Torres-Rodríguez, A., & Carbonell, X. (2015). Adicción a los videojuegos en línea: Tratamiento mediante el programa PIPATIC. *Aloma, 33*(2), 67–75.

[CR73] Torres-Rodriguez A, Carbonell X (2017). Update and proposal of treatment for Internet Gaming Disorder: PIPATIC program. Revista de Psicoterapia.

[CR74] Torres-Rodriguez, A., Griffiths, M. D., & Carbonell, X. (2017). Internet gaming disorder treatment: A case study evaluation of four adolescent problematic gamers. Manuscript submitted for publication.

[CR75] van Rooij AJ, Kuss DJ, Griffiths MD, Shorter GW, Schoenmakers MT, Van De Mheen D (2014). The (co-)occurrence of problematic video gaming, substance use, and psychosocial problems in adolescents. Journal of Behavioral Addictions.

[CR76] Williams D, Yee N, Caplan SE (2008). Who plays, how much, and why? Debunking the stereotypical gamer profile. Journal of Computer-Mediated Communication.

[CR77] Winkler A, Dörsing B, Rief W, Shen Y, Glombiewski JA (2013). Treatment of internet addiction: a meta-analysis. Clinical Psychology Review.

[CR78] Wittek, C. T., Finserås, T. R., Pallesen, S., Mentzoni, R. A., Hanss, D., Griffiths, M. D., & Molde, H. (2015). Prevalence and predictors of video game addiction: a study based on a national representative sample of gamers. *International Journal of Mental Health and Addiction*. 10.1007/s11469-015-9592-8. 10.1007/s11469-015-9592-8 PMC5023737 27688739

[CR79] Wölfling, K., Beutel, M. E., Dreier, M., & Müller, K. W. (2014). Treatment outcomes in patients with Internet addiction: a clinical pilot study on the effects of a cognitive-behavioral therapy program. *BioMed Research International, 2014*. 10.1155/2014/425924 PMC4100341 25097858

[CR80] Wood RTA, Griffiths MD, Chappell D, Davies MNO (2004). The structural characteristics of video games: a psycho-structural analysis. CyberPsychology & Behavior.

[CR81] Yee N (2006). The demographics, motivations and derived experiences of users of massively multi-user online graphical environments. Presence Teleoperators and Virtual Environments.

[CR82] Young KS (2007). Cognitive behavior therapy with Internet addicts: treatment outcomes and implications. CyberPsychology & Behavior.

[CR83] Young K (2009). Understanding online gaming addiction treatment issues for adolescents. The American Journal of Family Therapy.

[CR84] Young KS (2013). Treatment outcomes using CBT-IA with Internet-addicted patients. Journal of Behavioral Addictions.

